# Pharmacokinetics and absorption mechanism of tandospirone citrate

**DOI:** 10.3389/fphar.2023.1283103

**Published:** 2023-11-06

**Authors:** Rong Li, Yuwen Chen, Mi Jia, Xuehua Jiang, Ling Wang

**Affiliations:** ^1^ Antibiotics Research and Re-evaluation Key Laboratory of Sichuan Province, Sichuan Industrial Institute of Antibiotics, School of Pharmacy, Chengdu University, Chengdu, Sichuan, China; ^2^ Department of Clinical Pharmacy and Pharmacy Administration, Key Laboratory of Drug-Targeting and Drug Delivery System of the Education Ministry, West China School of Pharmacy, Sichuan University, Chengdu, Sichuan, China; ^3^ West China Hospital, West China School of Nursing, Sichuan University, Chengdu, Sichuan, China

**Keywords:** tandospirone citrate, pharmacokinetic, absorption mechanism, Caco-2 cell model, everted gut sac

## Abstract

Tandospirone citrate (TDS) is commonly used for the treatment of patients with generalized anxiety disorder in clinical practice, and several studies are developing new indications for TDS. However, the *in vivo* processes and absorption properties of TDS have not been systematically investigated. In this work, we conducted a comprehensive investigation using *in vivo*, *in vitro*, and *ex vivo* approaches, involving animal and cellular models, to examine the pharmacokinetic properties and absorption mechanisms of TDS. The results of *in vivo* studies revealed that the half-life (*t*_1/2_) of TDS was 1.380 ± 0.46 h and 1.224 ± 0.39 h following intragastric (i.g.) and intravenous (i.v.) administration of 20 mg/kg TDS, respectively. This indicates that TDS is rapidly eliminated in rats. The area under the curve (AUC) of TDS after i.g. and i.v. administration was 114.7 ± 40 ng/mL*h and 48,400 ± 19,110 ng/mL*h, respectively, and the absolute bioavailability of TDS was found to be low (0.24%). Furthermore, TDS was extensively metabolized in rats, with the AUC of the major active metabolite [1-[2-pyrimidyl]-piperazine] being approximately 16.38-fold higher than that of TDS after i.g. administration. The results from the *in vitro* Caco-2 cell model and *ex vivo* everted gut sac experiment demonstrated that TDS exhibited good permeability, and its transport was influenced by concentration, temperature, and pH. Passive diffusion was identified as the main absorption mechanism. In conclusion, TDS is classified as a Biopharmaceutics Classification System (BCS) class I drug, characterized by high solubility and permeability. The low absolute bioavailability of TDS may be attributed to its rapid metabolism. The pharmacokinetic data and absorption characteristics obtained in this study provide fundamental information for the further development and utilization of TDS.

## 1 Introduction

Tandospirone citrate (TDS), a partial agonist of the 5-HT1A receptor, is widely used in China and Japan for the treatment of patients with anxiety disorders caused by various neuroses, as well as anxiety states associated with physical diseases. TDS was included in China’s National Essential Drugs List in 2018, highlighting its importance in clinical practice. Its therapeutic benefits extend beyond anxiety and encompass other central nervous system (CNS) disorders such as Alzheimer’s disease, Parkinson’s disease, and schizophrenia (Huang et al., 2017). As our understanding of the underlying mechanisms of TDS in treating patients with these conditions deepens, its potential utilization in the treatment of patients with various CNS disorders is expected to increase. Recently, several studies have been exploring additional indications for TDS, including its use in vascular depression patients with mild cognitive impairment (Chen et al., 2023) and functional dyspepsia with anxiety (Liu et al., 2022). TDS can also be combined with other drugs to enhance their effects or alleviate adverse effects. For example, it has been shown to enhance the anti-myocardial fibrosis effect of valsartan in spontaneously hypertensive rats (Huang et al., 2020) and attenuate the respiratory depression caused by fentanyl (Fan et al., 2022). These findings highlight the versatility and potential of TDS in combination therapy. Despite its promising applications, the *in vivo* processes and absorption characteristics of TDS have not been systematically investigated, which may impede its further development. Understanding the pharmacokinetic properties and absorption mechanisms of TDS is crucial for optimizing its therapeutic use and exploring new indications.

Absolute bioavailability is a crucial parameter for evaluating the extent of drug absorption and utilization efficiency *in vivo*. It guides the selection of administration routes and optimization of drug dosage forms, ultimately improving drug efficacy. In addition, understanding the absorption characteristics of a drug is essential for various aspects of drug development and application, including drug delivery route selection, formulation design, prescription screening, process optimization, and clinical use. Therefore, the aim of this study is to investigate the absolute bioavailability and absorption mechanism of TDS, providing data support for its further development and utilization. The major active metabolite of TDS is 1-[2-pyrimidyl]-piperazine (1-PP) (Iyer et al., 2012; Hu et al., 2019). Initially, we examined the absolute bioavailability of TDS in rats and comprehensively assessed the pharmacokinetic characteristics of both TDS and 1-PP. Subsequently, the absorption characteristics of TDS were further investigated using *in vitro* and *ex vivo* methods. Because the permeability of drugs in Caco-2 monolayer cells correlates well with gastrointestinal absorption in humans, making it a gold standard for predicting drug absorption (Chen et al., 2016; Liu et al., 2019; Zhang et al., 2021), we employed the Caco-2 cell model to study the transport mechanism of TDS in this study. Furthermore, the *ex vivo* everted gut sac method is simple, rapid, and cost-effective, and it is often utilized to predict drug permeability and evaluate the impact of transporters on drug absorption (Al et al., 2012). Therefore, we employed the rat *ex vivo* everted gut sac method to further investigate the absorption characteristics of TDS.

In summary, this study comprehensively investigated the pharmacokinetic properties and absorption characteristics of TDS using various approaches, including *in vivo*, *in vitro*, and *ex vivo* studies, as well as animal and cellular models. The results will provide valuable insights for selecting the appropriate administration route, developing dosage forms, combining TDS with other drugs, and making informed decisions regarding its clinical application, especially when exploring new indications for TDS.

## 2 Materials and methods

### 2.1 Drugs and reagents

TDS was provided by Sichuan Keruide Kaihua Pharmaceutical Co. Ltd. 1-PP was supplied by J&K. Dapoxetine (DAP) was purchased from Taizhou Haichen Pharmaceutical Co., Ltd. Atenolol (ATE) was purchased from Dalian Meilun Biotechnology Co., Ltd. Propranolol hydrochloride (PRO) was provided by National Institutes for Food and Drug Control. Benzoic acid (BA) and rifampicin (RFP) were purchased from Adamas. Hank’s balanced salt solution (HBSS) was purchased from Beyotime Biotechnology. MTT was purchased from J&K Scientific. Urethane was purchased from Shanghai Yuanye Bio-Technology Co., Ltd.

### 2.2 Cells and animals

Caco-2 cells were obtained from the Center for Excellence in Molecular Cell Science, Chinese Academy of Sciences, and stored at West China School of Pharmacy, Sichuan University. The cells were cultured in Dulbecco’s modified Eagle medium and maintained in an incubator at 37°C with 5% CO_2_.

Sprague–Dawley (SD) rats (specific pathogen free, male, 6–8 weeks old) were purchased from Chengdu Dossy Experimental Animals Co., Ltd. The animals were quarantined in the animal facility at the West China School of Pharmacy, Sichuan University in Chengdu, China.

### 2.3 HPLC-MS/MS method for the determination of TDS and 1-PP

By adjusting the chromatographic conditions and mass spectrometry parameters, HPLC-MS/MS with high sensitivity and accuracy was established for the simultaneous determination of TDS and its main active metabolite 1-PP. We selected DAP as the internal standard (IS) due to its availability and cost-effectiveness. The HPLC-MS/MS analysis was performed using a CAPCELL PAK C18 column (5 μm, 200 mm I.D. × 50 mm) with a mobile phase for the binary gradient consisting of acetonitrile-water (containing 0.2% formic acid, V/V). The flow rate was set at 0.4 mL/min, the column temperature was maintained at 40°C, and the injection volume was 10 μL. The gradient elution program of the mobile phase is provided in Supplementary Table S1, and the mass spectrum parameters can be found in Supplementary Table S2. We validated the detection ability of the samples in plasma, Krebs–Ringer (K-R) buffer, and HBSS.

### 2.4 Sample preparation

On the day of analysis, acetonitrile was used in a 4:1 ratio with the sample to either precipitate plasma proteins or dilute the sample. After centrifugation at 12,000 rpm for 10 min, the organic layer was separated, filtered, and transferred into a vial for injection into the HPLC-MS/MS system.

### 2.5 Pharmacokinetic properties in rats

Twelve male SD rats were randomly divided into two groups: the i.g. group and the i.v. group. TDS was prepared in saline (0.9% NaCl aqueous solution), and both groups were administered TDS at a dose of 20 mg/kg. Blank blood samples were collected at 0 min before administration, and blood samples were collected at 2, 5, 8, 12, 20, 30, and 45 min, at 1, 2, 4, 7, and 10 h after administration. During the sampling process, rats were allowed to drink freely and food was provided 2 h after drug administration. The collected blood samples were placed in heparinized centrifuge tubes and centrifuged at 4,000 rpm for 10 min, and the plasma was separated and stored at −70°C. The plasma drug concentration was determined using HPLC-MS/MS, and the blood drug concentration–time data were fitted using DAS 3.0 pharmacokinetic software. Subsequently, the pharmacokinetic parameters of the i.g. group and the i.v. group were obtained. The absolute bioavailability was calculated using equation (Eq.) 1. AUC_i.g._ represents the area under the drug–time curve after i.g. administration and AUC_i.v_. represents the area under the drug–time curve after i.v. administration. D_i.g._ represents the dose administered in the i.g. group and D_i.v_. represents the dose administered in the i.v. group.

$$F_{\text{abs}}=\frac{{{\text{AUC}}_{i.g.}\times D}_{i.v.}}{{\text{AUC}}_{i.v.}\times D_{i.g.}}\times 100\%.$$ (1)

### 2.6 Growth inhibition assay

When Caco-2 cells reached 80% confluence, they were seeded in 96-well cell culture plates at a density of 1×10^4^ cells per well. After 24 h of culture, the cells were fully attached. The medium was then removed, and the drug-containing medium was added. Control wells without the drug-containing medium and blank wells without cells were also prepared. The cells were incubated for 12 h at 37°C in a 5% CO_2_ cell incubator. After incubation, the medium was discarded, and the cells were carefully washed three times with PBS. Subsequently, 200 μL of 0.5% MTT solution was added to each well in the dark, and the culture was continued for 4 h. The culture medium was then gently aspirated, and 100 μL of DMSO was added to each well. The cells were placed on a low-speed shaker for 10 min, and the absorbance value at 490 nm was measured using a microplate reader.

The absorbance value at 490 nm for each well was recorded as A, and the cell inhibition rate was calculated according to Eq. 2. A_0_ represents the OD value of the zeroed wells, and A_Control_ represents the OD value of the control wells.

$$\text{Inhibition rate}\left(\%\right)=\left[1-\left(A_{\text{Test}\;\text{drug}}-A_{0}\right)/\left(A_{\text{Control}}-A_{0}\right)\right]\times 100.$$ (2)

### 2.7 Validation of tight junction integrity in the Caco-2 cell model

Caco-2 cells in the logarithmic growth phase were seeded onto 24-well polyester clear Transwell^®^ inserts with a membrane area of 0.33 cm^2^ and a pore size of 0.4 μm at a density of 10^5^ cells/cm^2^ per well (Wen et al., 2021; Tao et al., 2022). Apical (AP) and basolateral (BL) compartments of the transport plate were filled with 0.2 mL and 1 mL of liquid, respectively. The cells were then incubated at 37°C in a 5% CO_2_ incubator, and the culture medium was changed every other day. The resistance values of the cells were monitored at 4, 6, 11, 16, 19, 21, and 23 days after seeding using an epithelial tissue voltohmmeter (EVOM2, World Precision Instruments, Sarasota, FL, United States).

The trans-epithelial electrical resistance (TEER) was calculated according to Eq. 3, where *R*_T_ is the measured resistance value, *R*_0_ is the blank resistance value, and *A* is the polycarbonate film area.

$$\text{TEER}=\left(R_{T}-R_{0}\right)*A.$$ (3)

### 2.8 Caco-2 cell transport assay

The 24-well Transwell® inserts with a TEER value greater than 350 Ω-cm^2^ were selected for the transport experiment, (Shen et al., 2015a; Liu et al., 2017). The Transwell® inserts were washed twice with HBSS and incubated with blank HBSS for 30 min before the test drugs were added. For apical-to-basolateral (AP-BL) transport, 0.2 mL of the test drug was added to the AP side as the donor chamber and 1 mL of blank HBSS was added to the BL side as the receiver chamber. For basolateral-to-apical (BL-AP) transport, 1 mL of the test drug was added to the BL side as the donor chamber and 0.2 mL of blank HBSS was added to the AP side as the receiver chamber. The 24-well Transwell® inserts were then placed in a 37°C shaker at 50 r/min. Samples of 0.05 mL were collected from the receiver chamber at 30, 60, 90, and 120 min, and 0.05 mL of blank HBSS was added at the same time.

To investigate the effect of temperature on the bidirectional transport of TDS, the experimental group was placed in 4°C (Rocha et al., 2013). When examining the effect of pH on the bidirectional transport of TDS, the HBSS at pH 7.4 was adjusted to pH 5.5 in the experimental group (Wu et al., 2020). To examine the effect of transporters on the bidirectional transport of TDS, inhibitors were added to the donor chamber 30 min in advance in the experimental group, and then washed with HBSS.

The uptake (Qi) and the apparent permeability coefficient (*P*_app_) (cm·s^−1^) were calculated according to Eqs. 4 and 5, respectively. *C* *_i_* represents the concentration at the sampling time point in the receiving chamber, while *C* *_0_* represents the initial concentration in the donor chamber.

$$Q_{i\left(\text{AP}-\text{BL}\right)}=0.05\times \sum_{i=1}^{n}C_{i-1}+1.0\times C_{i},$$ (4)

$$P_{\text{app}}=\left(\left.dQ/dt\right)/\left(A*\right.C_{0}\right).$$ (5)

### 2.9 Drug absorption in *ex vivo* everted gut sacs

According to Yu et al. (2019) and Hu et al. (2023), an *ex vivo* everted gut sacs experiment was conducted. Rats were anesthetized with intraperitoneal injection of 20% urethane (5 mL/kg). The abdominal cavity was opened along the midline of the abdomen, approximately 3 cm, and a section of the small intestine (approximately 10 cm) was quickly clipped. The animals were then euthanized by cervical dislocation. The intestinal tube was rinsed with iced normal saline until no content was flowing out. One end of the intestinal tube was ligated with a surgical thread, and then the tube was inverted using a glass rod. After cleaning with the K-R buffer (pH 7.4), the other end was ligated with a plastic tube to form an intestinal sac. In total, 1 mL of the K-R buffer at 37°C was injected into the intestinal sac, which was then placed in the blank K-R buffer and equilibrated for 5 min. Subsequently, the sac was transferred to a drug-containing K-R buffer. The experiment was conducted at a temperature of 37°C, and a gas mixture of 95% O_2_ and 5% CO_2_ was passed into the test solution. Samples of 100 μL were collected from the intestinal sac at 0, 30, 45, 60, 90, and 120 min and replenished with the same volume of the blank K-R buffer.

At the end of the experiment, the length (l) and circumference (c) of the intestinal segment were measured to calculate the surface area (*A*). The cumulative absorption amount (Q) (Eq. 6) and the apparent permeability coefficient (*P*_app_) (Eq. 5) were calculated.

$$Q=1\times C_{n}+0.1\times \sum_{i=1}^{n-1}C_{i}.$$ (6)

### 2.10 Statistical analysis

All experimental data were expressed as mean ± standard deviation. The data were processed using Prism 8.0 statistical software, and statistical analysis was performed by *t*-test or one-way analysis of variance (ANOVA). A significance level of *p* < 0.05 was considered statistically significant. The following notation was used for indicating levels of significance: *p < 0.05, **p < 0.01, and ***p < 0.001.

## 3 Results and discussion

### 3.1 HPLC-MS/MS method for the determination of TDS and its major active metabolite 1-PP

There are limited methods for simultaneous detection of TDS and its major active metabolite 1-PP. Although the existing HPLC-MS/MS method exhibits good sensitivity and selectivity, its practical application is somewhat restricted due to the requirement of expensive TDS-d8 and 1-PP-d8 as internal standards (Hu et al., 2019). In this study, we established a highly sensitive and selective HPLC-MS/MS method that offers a wider linear range, faster detection speed, and compatibility with various biological matrices. To overcome the limitations of cost and availability, we utilized the inexpensive and easily accessible DAP as the internal standard.

**FIGURE 1:**
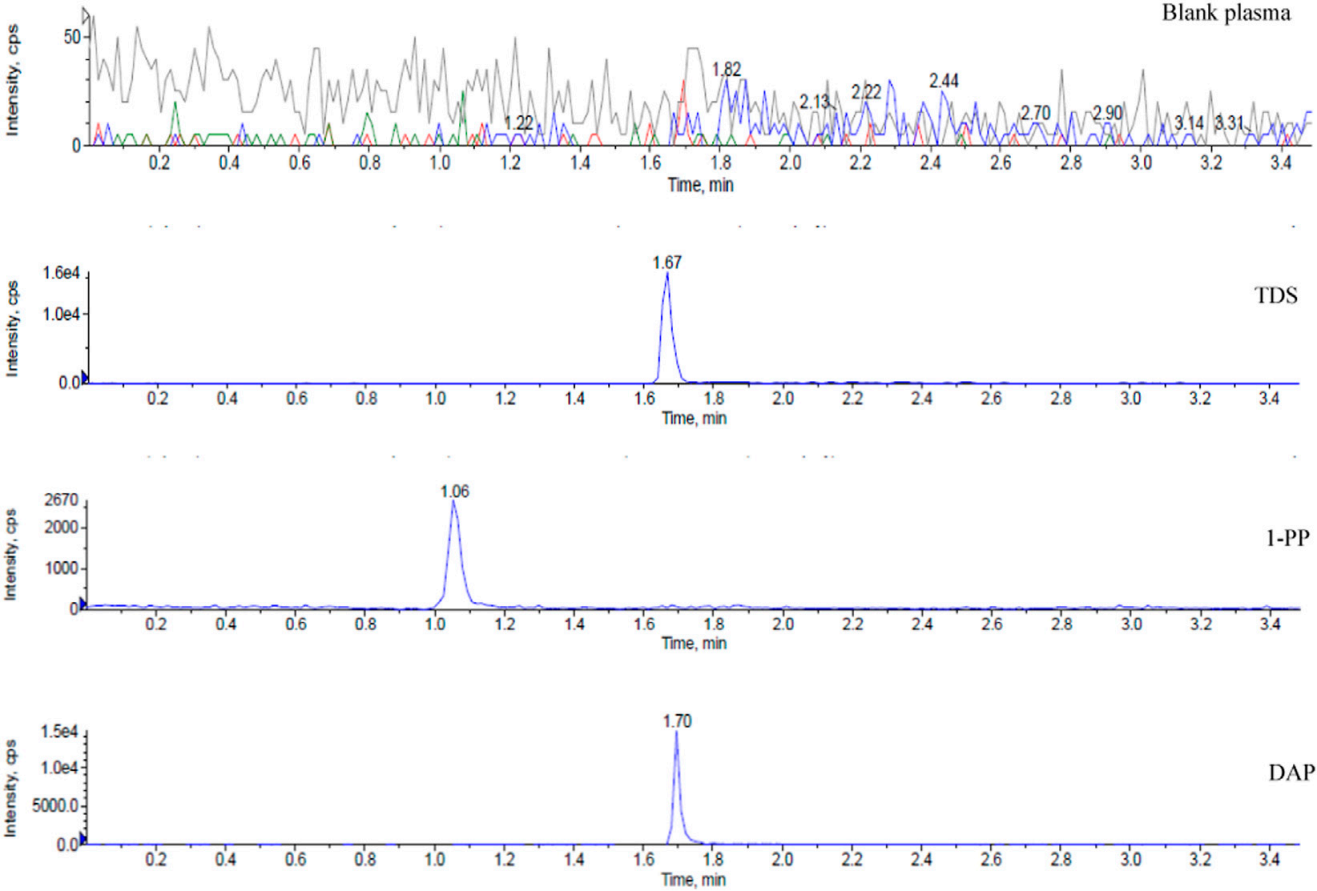
Representative chromatograms of blank, TDS, 1-PP, and DAP in the plasma sample.

Representative chromatograms are presented in Figure 1, demonstrating the retention times of TDS, 1-PP, and the internal standard DAP in rat plasma at approximately 1.67 min, 1.06 min, and 1.70 min, respectively. Impurities in the tissue did not interfere with the peaks of the analytes or the internal standard. Chromatographic separation was accomplished within 3.5 min for each sample. The linear range of TDS in rat plasma was determined to be 2.024–1,012 ng/mL (low concentration) and 406.4–243800 ng/mL (high concentration). For 1-PP, the linear range was 10–1,000 ng/mL. This method also exhibited excellent sensitivity and selectivity when applied to plasma, cell, and intestinal fluid samples.

### 3.2 Pharmacokinetic properties of TDS in rats

**FIGURE 2:**
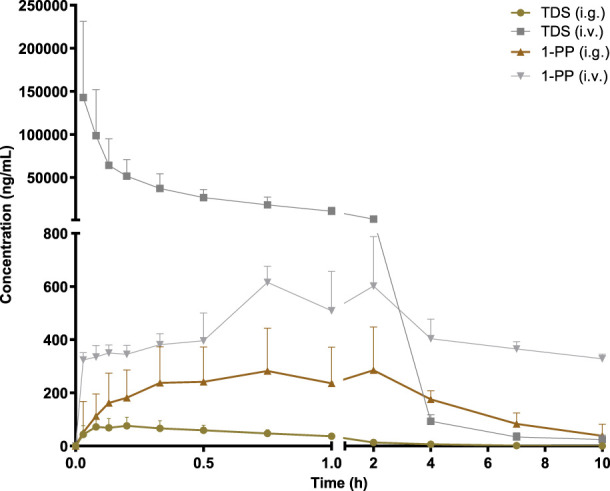
Average plasma concentration–time curve in rats after i.g. and i.v. 20 mg/kg of TDS (*n* = 6).

The pharmacokinetic data of TDS play a crucial role in the further development and utilization of this drug. As the major active metabolite of TDS, simultaneous measurement of plasma concentrations of TDS and 1-PP was conducted in this study. The plasma concentration–time curves of TDS and 1-PP in rats following i.g. and i.v. administration were fitted and depicted in Figure 2. Pharmacokinetic parameters were calculated using DAS 3.0 pharmacokinetic software and are presented in Table 1.

**TABLE 1:** Main pharmacokinetic parameters of TDS and 1-PP in rats (*n* = 6).

Parameter	Unit	TDS		1-PP	
		i.g.	i.v.	i.g.	i.v.
AUC_(0-t)_	ng/mL*h	114.1 ± 41	48,375 ± 19,097	1,512 ± 238.4	4,201 ± 476
AUC_(0-∞)_	ng/mL*h	114.7 ± 41	48,397 ± 19,107	1879 ± 616.2	17,979 ± 12,674
MRT_(0-t)_	h	1.534 ± 0.30	0.516 ± 0.08	3.546 ± 1.19	4.523 ± 0.22
*t*_1/2_	h	1.380 ± 0.46	1.224 ± 0.39	3.497 ± 3.64	29.498 ± 28.34
*t*_max_	h	0.161 ± 0.09	-	2.25 ± 1.47	1.3 ± 0.65
Vz/F	L/kg	412.841 ± 240.59	0.841 ± 0.42	47.261 ± 28.17	39.392 ± 14.47
CLz/F	L/h/kg	194.016 ± 69.01	0.476 ± 0.19	11.352 ± 2.64	1.686 ± 1.10
C_max_	ng/mL	83.23 ± 35.36	144,850 ± 86,523	357.2 ± 135.5	691.0 ± 82

The results for TDS revealed that *t*_max_ was 0.161 ± 0.09 h after i.g. administration. *t*_1/2_ of TDS was determined to be 1.380 ± 0.46 h and 1.224 ± 0.39 h after i.g. and i.v. administration, respectively. These indicated rapid absorption and elimination of TDS. The AUC_(0-∞)_ values of TDS after i.g. and i.v. administration were 114.7 ± 41 ng/mL*h and 48,397 ± 19,107 ng/mL*h, respectively. The absolute bioavailability of TDS was calculated to be 0.24%. In addition, the AUC_(0-∞)_ of 1-PP was approximately 16.38-fold higher than that of TDS after i.g. administration, indicating extensive metabolism of TDS in rats. According to Natsui et al. (2007) and Iyer et al. (2012), TDS undergoes abundant metabolic pathways *in vivo*, primarily mediated by CYP3A4, with 1-PP being its primary active metabolite. Due to its significant hepatic first-pass effect, its absolute bioavailability is very low. In addition, TDS was rapidly eliminated *in vivo* due to first-pass elimination in the liver, resulting in a short half-life. On the other hand, 1-PP had a longer half-life, and no significant first-pass effect was observed. Therefore, close attention should be paid to the pharmacological effects of 1-PP when exploring new indications for TDS.

### 3.3 Growth inhibition assay of Caco-2 cells

**FIGURE 3:**
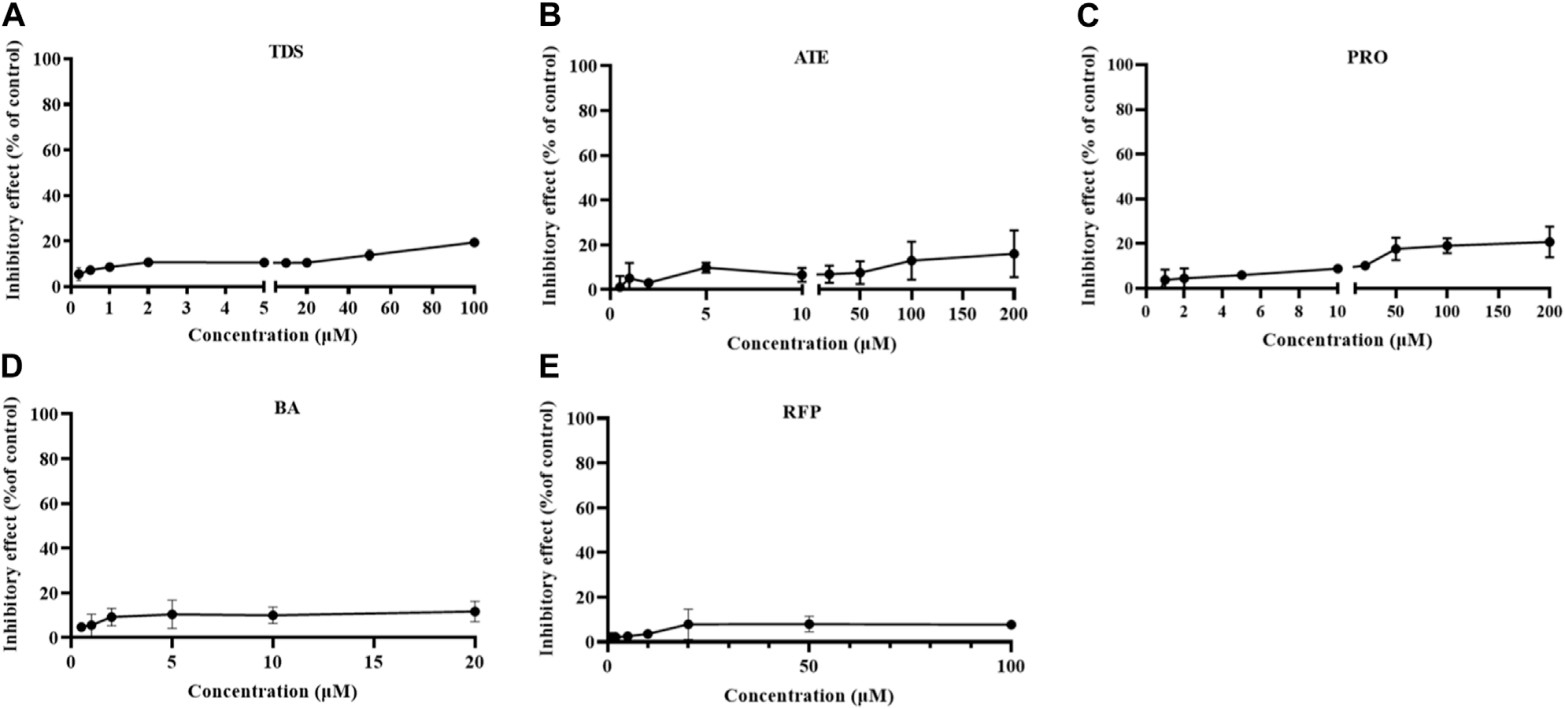
Growth inhibition rates of Caco-2 cells treated by **(A)** TDS, **(B)** ATE, **(C)** PRO, **(D)** BA, and **(E)** RFP (*n* = 3).

The growth inhibition of drugs on the cells needs to be clear before carrying out the cell transport assay. Cells were treated with 0∼100 μmol·L^−1^ TDS, 0∼200 μmol·L^−1^ ATE, 0∼200 μmol·L^-1^ PRO, 0∼20 mmol·L^−1^ BA, and 0∼100 μmol·L^−1^ RFP for 12 h. The results in Figure 3 show that the inhibition rates of TDS, ATE, PRO, BA, and RFP at the corresponding concentrations were less than 20% on Caco-2 cells. It indicated that the appropriate concentration of drugs could be selected for the transporter experiment.

### 3.4 Validation of the Caco-2 cell transport model

**FIGURE 4:**
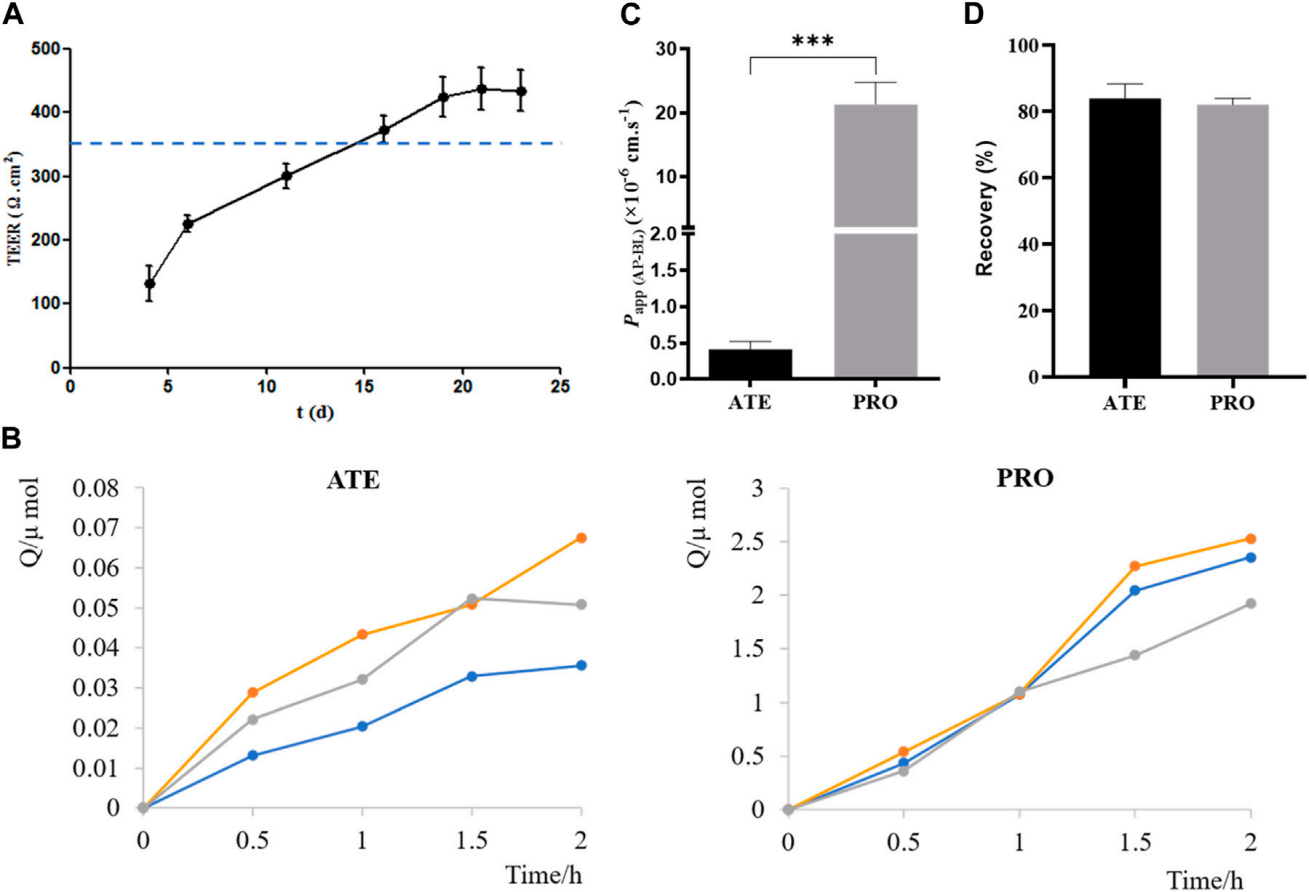
Validation of the Caco-2 cell transport model. **(A)** TEER values of Caco-2 cells on 4, 6, 11, 16, 19, 21, and 23 days after seeding plates. **(B)** Cumulative drug absorption of ATE and PRO. **(C)** *P*_app (AP-BL)_ of ATE and PRO. **(D)** Recovery of ATE and PRO in the transport assay (*n* = 3).

Before carrying out the transport experiment, it is necessary to verify the function of model cells, that is, the tight junction of Caco-2 cells. The transmembrane resistance value of cells on the polycarbonate membrane was measured by EVOM. When TEER was greater than or equal to 350 Ω·cm^2^, the cells formed tight junctions (Shen et al., 2015a; Liu et al., 2017). As shown in Figure 4A, TEER values exceeded 350 Ω·cm^2^ from days 16 to 23 of inoculation, indicating that Caco-2 cells formed tight junctions on polycarbonate membranes, and they could be used for transport experiments.

ATE and PRO are commonly used as model drugs for low and high permeability. When *P*_app_ of ATE and PRO in Caco-2 cells is approximately 10^–7^ cm·s^−1^ and 10^–5^ cm·s^−1^, respectively, it proves that there is a dense tight junction between cells, which can provide reference for the permeability of test drugs in cells (Smetanová et al., 2011; Wang and Yang, 2017; Luo et al., 2018). Here, the results of the transport experiment showed a good linear relationship between the cumulative uptake and the uptake time, which could be used for the calculation of *P*_app_ (Figure 4B). *P*_app (AP-BL)_ of ATE (50 μmol·L^−1^) was (4.1 ± 0.11) ×10^–7^ cm·s^−1^ and that of PRO (5 μmol·L^−1^) was (2.1 ± 3.44) ×10^−5^ cm·s^−1^ (Figure 4C), which was consistent with the reported values in the literature, indicating that the Caco-2 cell model in this study formed dense tight junctions. It can be used for subsequent bidirectional transport studies. In addition, the drug recovery rates during the transport experiment were verified, and the average recovery rates were more than 80% (Figure 4D), indicating that the uptake of the drug by the cell monolayer and the evaporation of solvent basically had no effect on the experimental results, and *P*_app_ obtained in the experiment was accurate (Hubatsch et al., 2007).

### 3.5 Effect of concentration on bidirectional transport of TDS

**FIGURE 5:**
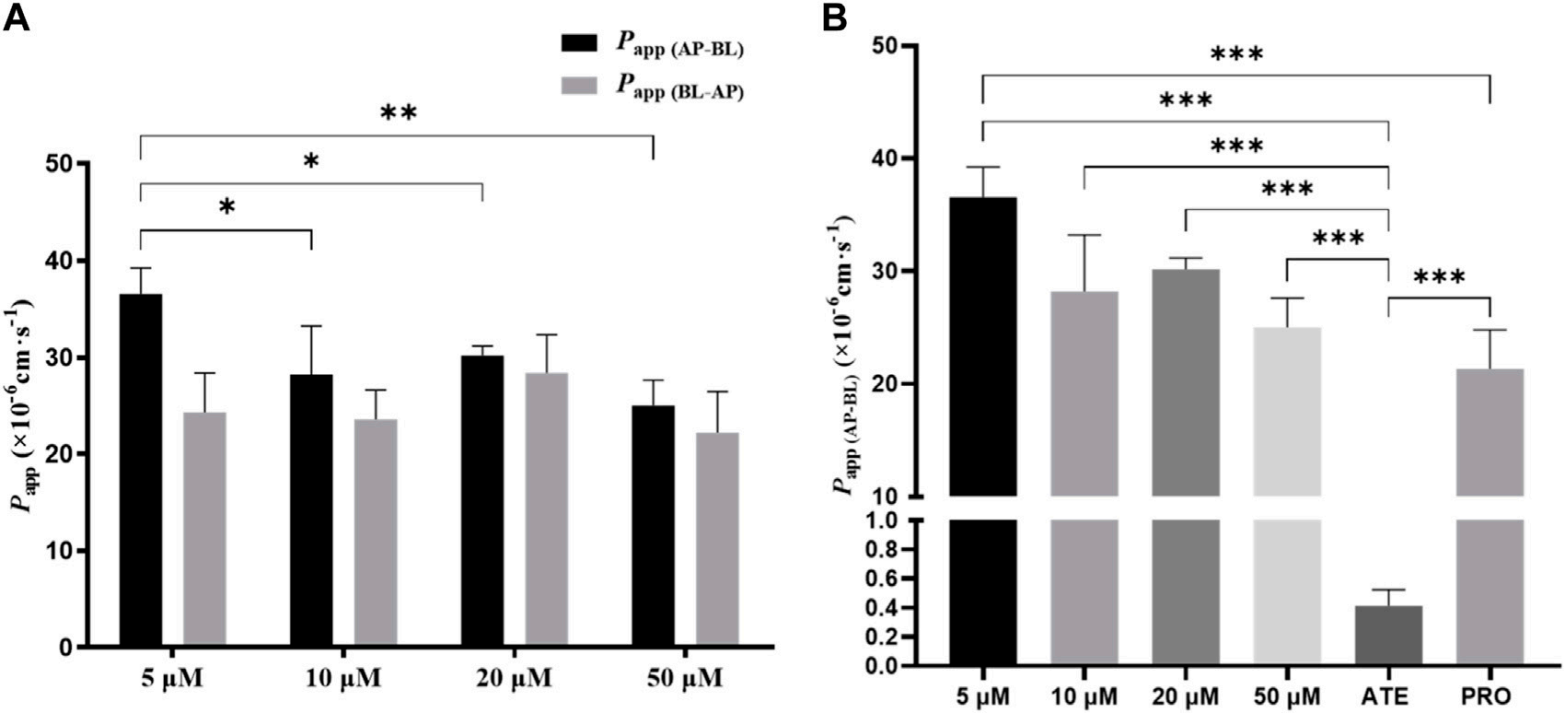
Effect of concentration on TDS transport. **(A)** *P*_app (AP-BL)_ and *P*_app (BL-AP)_ of TDS in Caco-2 cells at different concentrations. **(B)** *P*_app (AP-BL)_ of drugs compared with model drugs (*n* = 3).

To investigate whether the transport of TDS was concentration-dependent, combined with MTT results, 5 μmol·L^−1^, 10 μmol·L^−1^, 20 μmol·L^−1^, and 50 μmol·L^−1^ of TDS were selected for the bidirectional transport experiment. As shown in Figure 5A, there was no significant difference in *P*_app (AP-BL)_ for TDS in the concentration range of 10–50 μmol·L^−1^, whereas *P*_app (AP-BL)_ at 5 μmol·L^−1^ was significantly higher than the other three concentrations. All of *P*_app (BL-AP)_ were (20–30) ×10^−6^ cm·s^−1^, with no significant differences. Figure 5B shows that *P*_app (AP-BL)_ of 5 μmol·L^−1^ TDS was significantly higher than that of ATE and higher than that of PRO. Taken together, it is speculated that TDS has good permeability in Caco-2 cells and is similar to PRO. TDS had the best permeability at low concentrations, indicating that the transport of TDS was mainly passive diffusion, with uptake transporters also involved, but the effect of transporters was masked by passive diffusion at higher concentrations.

### 3.6 Effect of temperature on bidirectional transport of TDS

**FIGURE 6:**
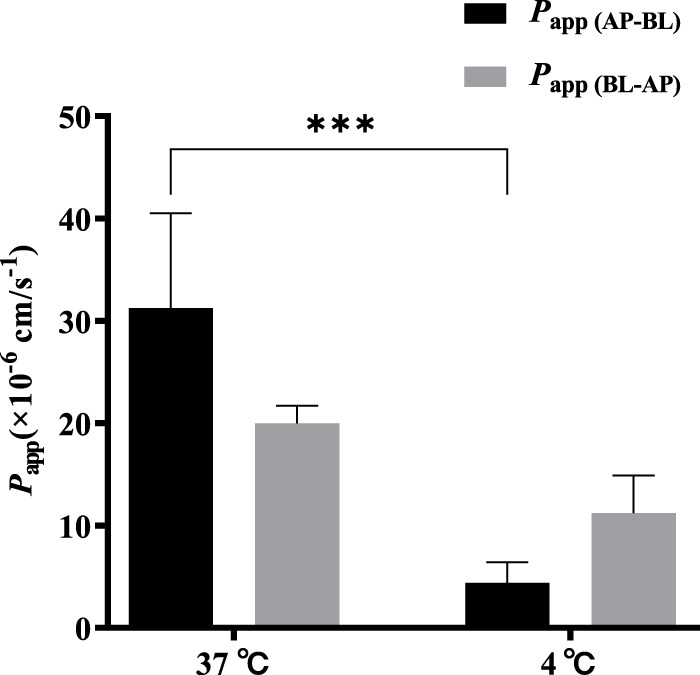
Effect of temperature on TDS transport. *P*_app (AP-BL)_ and *P*_app (BL-AP)_ of TDS in Caco-2 cells at 37 °C and 4 °C (*n* = 3).

A low concentration (5 μmol·L^−1^) of TDS was selected to investigate the effect of temperature on the bidirectional transport. Figure 6 shows that when the temperature was decreased from 37 °C to 4 °C, both *P*_app (AP-BL)_ and *P*_app (BL-AP)_ were significantly decreased, with a more dramatic decrease observed in *P*_app (AP-BL)_. These results indicated that the transport of TDS in Caco-2 cells was temperature-sensitive and an energy-dependent process, suggesting the involvement of transporters in the transport from AP to BL.

### 3.7 Effect of pH on bidirectional transport of TDS

**FIGURE 7:**
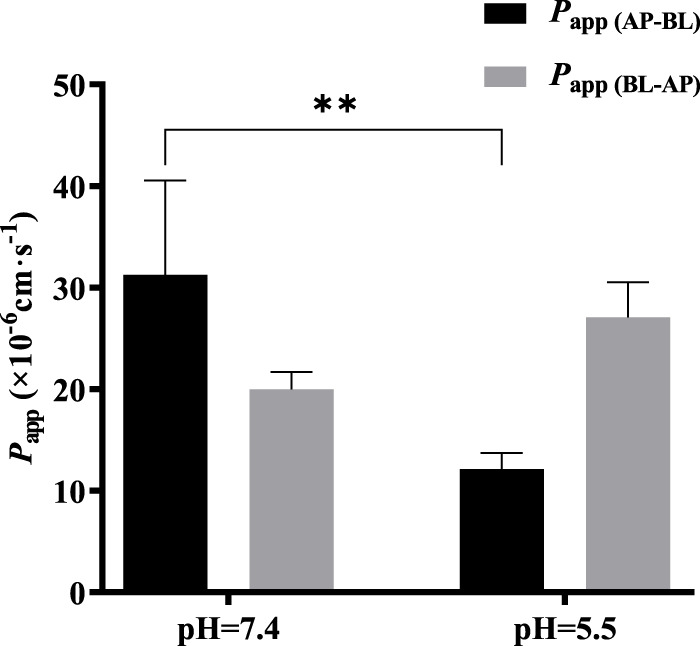
Effect of pH on TDS transport. *P*_app (AP-BL)_ and *P*_app (BL-AP)_ of TDS in Caco-2 cells at pH = 7.4 and pH = 5.5 (*n* = 3).

The effect of pH on the bidirectional transport of TDS was investigated by preparing 5 μmol·L^−1^ TDS in pH 5.5 HBSS. As shown in Figure 7, when the pH of the transport solution decreased from 7.4 to 5.5, there was a significant decrease in *P*_app (AP-BL)_, whereas *P*_app (BL-AP)_ showed no significant change. These results suggest that the transport of TDS in Caco-2 cells may be influenced by pH.

### 3.8 Effect of transporters on bidirectional transport of TDS

Based on the results obtained at different concentrations and temperatures, it can be speculated that uptake transporters may be involved in the transport of TDS. The uptake transporters on the villous side of human intestinal epithelial cells mainly include OATP, MCT1, PEPT1, and ASBT. Among them, OATP is involved in the transport of various drugs, including anticancer drugs, antibiotics, lipid-lowering drugs, glucose-lowering drugs, and toxins and poisons (International Transporter et al., 2010; Lee et al., 2017). In addition, there have been reports suggesting the involvement of MCT1 in the transport of TDS (Hobara et al., 2007). Therefore, we investigated the effect of OATP and MCT1 on the transport of TDS.

**FIGURE 8:**
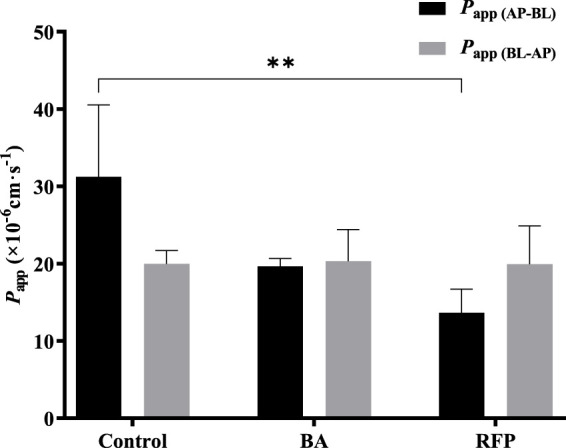
Effect of transporters on transport of TDS. *P*_app (AP-BL)_ and *P*_app (BL-AP)_ in Caco-2 cells when BA and RFP were added (*n* = 3).

BA (Kobayashi et al., 2004; Shen et al., 2015b) and RFP (Zhou et al., 2016; Xiang et al., 2021) are commonly used inhibitors of MCT1 and OATP, respectively. Referring to the literature works, we selected 10 mmol·L^−1^ BA and 50 μmol·L^−1^ RFP for the bidirectional transport experiments. Figure 8 shows that *P*_app (AP-BL)_ and *P*_app (BL-AP)_ in the BA group were not significantly different from those in the control group, indicating that the transport of TDS in Caco-2 cells may not be affected by the MCT1 transporter. There was no significant difference in *P*_app (BL-AP)_ between the RFP group and the control group, whereas *P*_app (AP-BL)_ was significantly reduced, suggesting that the transport of TDS from AP to BL in Caco-2 cells may be affected by OATP transporters. However, *P*_app (AP-BL)_ of TDS in the RFP group was still larger than 10 × 10^-6^ cm s^−1^, indicating that the effect of OATP transporters on the transmembrane transport of TDS was limited, and the main transport way for TDS was passive diffusion.

### 3.9 Absorption of TDS in the *ex vivo* everted gut sac

**FIGURE 9:**
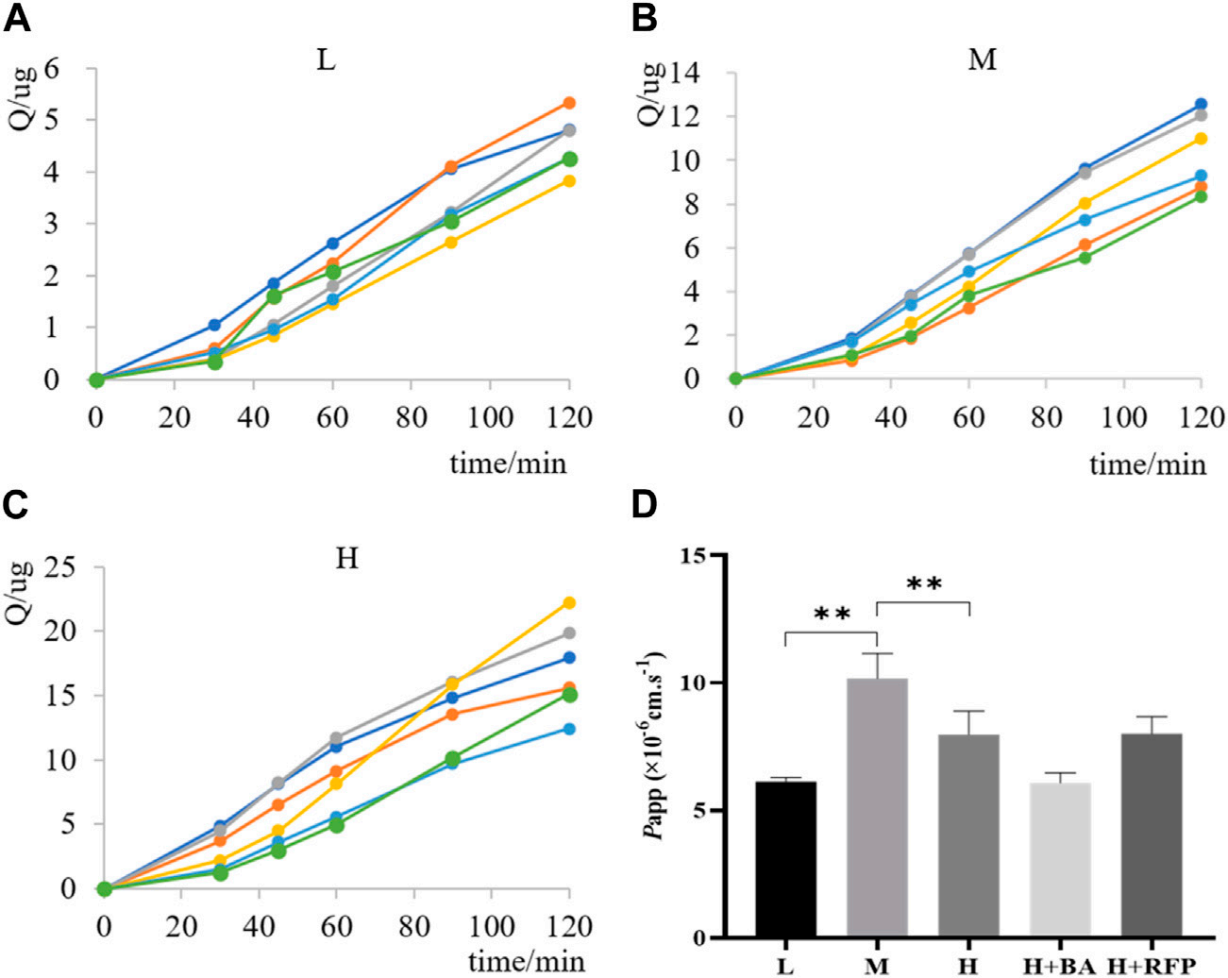
Transport of TDS in the everted gut sac. Permeability curves for **(A)** low (L) concentration, **(B)** medium (M) concentration, and **(C)** high (H) concentration. **(D)** *P*_app_ of TDS with L, M, and H concentrations and inhibitors (*n* = 6).

The *ex vivo* everted gut sac method is a classical approach for studying small intestinal absorption by quantitatively characterizing the permeability of drugs that pass through the intestinal mucosa. Therefore, we applied the everted gut sac method to investigate the intestinal absorption of TDS. The permeability rate curves of TDS at low, medium, and high concentrations were plotted by calculating the cumulative absorption, Q (Figures 9A–C). The curves showed good linearity (*R* ^2^ > 0.95), which allowed for the calculation of *P*_app_. Figure 9D demonstrates that *P*_app_ of TDS at the medium concentration was significantly higher than that at the low and high concentrations, whereas there was no significant difference between the low and high concentrations. These results suggested the possible involvement of transporters in the transport of TDS. However, when MCT1 and OATP inhibitors were added, the results did not significantly differ from *P*_app_ in the control group, indicating that MCT1 and OATP transporters may not be involved in the absorption of TDS.

From cellular experiments, it was determined that OATP is involved in the transport of TDS, whereas from everted gut sac experiments, it was found that OATP is not involved in the absorption of TDS. According to the Zhao (2016), there were species differences in the expression of OATP isoforms in human and rat intestines. Both human and rat intestines express OATP 2B1 (Oatp 2b1), whereas only rat intestines express Oatp 1a5. RFP can inhibit the expression of OATP 2B1 (Oatp 2b1) in the intestinal tract, but the regulatory role of Oatp 1a5 is not clear. Therefore, inconsistent results between cellular and animal experiments may be attributed to the inability of RFP to modulate the expression of Oatp 1a5, which is potentially involved in the absorption of TDS.

## 4 Conclusion

This work systematically investigated the *in vivo* process and absorption characteristics of TDS. The *in vivo* model examined the pharmacokinetic properties of TDS in rats and determined an oral bioavailability of approximately 0.24%. The results of the *in vitro* Caco-2 cell model and *ex vivo* everted gut sac method indicated that TDS exhibited good permeability in the intestine, with passive diffusion being the main mode of drug absorption. Overall, TDS was classified as a Biopharmaceutics Classification System (BCS) class I drug with high solubility and high permeability, but its oral bioavailability was low due to rapid and extensive metabolism in the body. Understanding the characteristics of TDS, such as high permeability and low oral bioavailability, will serve as a reference for future development, including the selection of administration routes, formulation development, combination drug use, and rational clinical application.

## Data Availability

The original contributions presented in the study are included in the article/Supplementary Material; further inquiries can be directed to the corresponding authors.
