# Supplementary material for: Pharmacokinetics and absorption mechanism of tandospirone citrate
Source: Front Pharmacol. 2023 Nov 6;14:1283103. doi: 10.3389/fphar.2023.1283103 (PMC10657815; doi:10.3389/fphar.2023.1283103)
Supplement: Supplementary file 1 [file Table1.DOCX]

**Supporting information**

**Table S1 Gradient elution program of mobile phase**

| *t*/min | Acetonitrile/% |
| --- | --- |
| 0.01 | 10 |
| 0.50 | 10 |
| 1.00 | 50 |
| 1.50 | 95 |
| 2.50 | 95 |
| 2.51 | 10 |
| 3.50 | Stop |

**Table S2 Parameters of mass spectrometry in the assays of TDS, 1-PP and DAP**

| Parameters | TDS | 1-PP | DAP |
| --- | --- | --- | --- |
| m/z (amu) | 384.3/122.2 | 165.1/122.3 | 306.0/260.8 |
| Nebulizer Gas (psi) | 15 | | |
| Curtain Gas (psi) | 6 | | |
| Collision Gas (psi) | 8 | | |
| Ionspray Voltage (V) | 5000 | | |
| Temperature (℃) | 400 | | |
| Declustering Potential (V) | 40 | 32 | 43 |
| Focusing Potential (V) | 117 | 95 | 148 |
| Entrance Potential (V) | 5 | 3 | 5 |
| Collision Energy (V) | 18 | 22 | 19 |
| Collision Cell Exit Potential (V) | 7 | 6 | 14 |

**Table S3 Results of accuracy and precision test in TDS and 1-PP plasma sample（i.g.） (mean ± sd, n=6)**

| Analytes | Conc.(ng/mL) | Recovery (%) | RSD _intra_(%) | RSD _inter_(%) |
| --- | --- | --- | --- | --- |
| TDS | 2.024 | 93.90 ± 14.01 | 4.08～7.51 | 14.92 |
|  | 4.048 | 96.98 ± 4.62 | 0.60～6.61 | 4.76 |
|  | 50.60 | 103.36 ± 5.66 | 1.60～6.73 | 5.26 |
|  | 809.6 | 99.67 ± 6.42 | 3.75～4.80 | 6.44 |
| 1-PP | 10.00 | 104.45 ± 9.04 | 8.16～11.14 | 8.96 |
|  | 20.00 | 97.05 ± 13.24 | 8.77～13.43 | 11.13 |
|  | 100.0 | 98.55 ± 11.33 | 11.24～13.42 | 12.21 |
|  | 800.0 | 104.68 ± 12.80 | 5.26～12.16 | 9.06 |

**Table S4 Results of accuracy and precision test in TDS and 1-PP plasma sample（i.v.） (mean ± sd, n=6)**

| Analytes | Conc.(ng/mL) | Recovery (%) | RSD _intra_(%) | RSD _inter_(%) |
| --- | --- | --- | --- | --- |
| TDS | 406.4 | 98.48 ± 11.34 | 9.61～10.53 | 12.22 |
|  | 1016 | 97.32 ± 9.58 | 7.67～9.92 | 9.27 |
|  | 12190 | 94.70 ± 4.10 | 3.05～4.34 | 5.55 |
|  | 195000 | 99.53 ± 4.01 | 2.51～4.03 | 3.23 |
| 1-PP | 10.00 | 110.50 ± 12.20 | 5.85～11.04 | 14.23 |
|  | 20.00 | 106.80 ± 13.75 | 7.78～12.90 | 10.33 |
|  | 100.0 | 104.93 ± 10.51 | 3.05～9.84 | 7.59 |
|  | 800.0 | 100.32 ± 7.80 | 7.95～9.12 | 8.97 |

**Table S5 Results of recovery test in plasma（mean ± sd, n=6）**

| Analytes | Conc.(ng/mL) | Recovery (%) |
| --- | --- | --- |
| TDS (i.g.) | 4.048 | 78.27 ± 0.96 |
|  | 50.60 | 76.10 ± 2.49 |
|  | 809.6 | 78.96 ± 4.81 |
| TDS (i.v.) | 1016 | 76.33 ± 12.05 |
|  | 12190 | 77.87 ± 13.74 |
|  | 195000 | 84.16 ± 9.74 |
| 1-PP | 0.02000 | 63.74 ± 11.12 |
|  | 100.0 | 70.63 ± 6.49 |
|  | 800.0 | 82.83 ± 11.82 |
| IS | 5733 | 87.41 ± 8.93 |
|  | 22930 | 116.43 ± 5.53 |

**Table S6 Results of matrix effect test in plasma. (mean ± sd, n=6)**

| Analytes | Conc. (ng/mL) | IS-normalized MF | RSD (%) |
| --- | --- | --- | --- |
| TDS (i.g.) | 4.048 | 0.27 ± 0.02 | 8.53 |
|  | 809.6 | 0.23 ± 0.02 | 7.90 |
| TDS (i.v.) | 1016 | 0.68 ± 0.04 | 10.20 |
|  | 195000 | 0.76 ± 0.05 | 6.19 |
| 1-PP | 20.00 | 1.01 ± 0.06 | 5.94 |
|  | 800.0 | 0.96 ± 0.07 | 7.29 |
| IS | 5733 | 1.02 ± 0.05 | 4.90 |
|  | 22930 | 1.07 ± 0.09 | 8.41 |

**Table S7 Results of stability test in plasma samples. (mean ± sd, n=6)**

| Analytes | Conc. (ng/mL) | Bias(%) | | |  |
| --- | --- | --- | --- | --- | --- |
|  |  | 6h (Room temperature) | 24h (4℃) | 10d (-70℃) | Ft (2t) |
| TDS (i.g.) | 4.048 | 1.38 | 14.16 | 7.50 | 14.50 |
|  | 809.6 | 1.60 | 11.76 | 7.35 | 1.25 |
| TDS (i.v.) | 1016 | 2.35 | 0.16 | 6.47 | 6.72 |
|  | 195000 | 5.19 | 1.87 | 8.34 | 3.40 |
| 1-PP | 20.00 | 2.92 | 0.86 | 10.42 | 8.63 |
|  | 800.0 | 1.23 | 3.62 | 5.39 | 0.04 |
